# Association of working shifts, inside and outside of healthcare, with severe COVID−19: an observational study

**DOI:** 10.1186/s12889-021-10839-0

**Published:** 2021-04-22

**Authors:** A. V. Rowlands, C. Gillies, Y. Chudasama, M. J. Davies, N. Islam, D. E. Kloecker, C. Lawson, M. Pareek, C. Razieh, F. Zaccardi, T. Yates, K. Khunti

**Affiliations:** 1 Diabetes Research Centre, Leicester General Hospital, University of Leicester, Gwendolen Rd, Leicester, LE5 4PW UK; 2 National Institute for Health Research (NIHR) Leicester Biomedical Research Centre (BRC), Leicester General Hospital, Leicester, LE5 4PW UK; 3 Leicester Real World Evidence Unit, Diabetes Research Centre, Leicester General Hospital, University of Leicester, Gwendolen Rd, Leicester, LE5 4PW UK; 4 Nuffield Department of Population Health, University of Oxford, Oxford, UK; 5 St George’s University of London, Tooting, London, UK; 6 Department of Respiratory Sciences, University of Leicester, Leicester, UK; 7 NIHR Applied Research Collaboration – East Midlands (ARC-EM), Leicester General Hospital, Leicester, UK

**Keywords:** Coronavirus, SARS-CoV-2, Employment, Ethnicity, UK Biobank

## Abstract

**Background:**

Health and key workers have elevated odds of developing severe COVID-19; it is not known, however, if this is exacerbated in those with irregular work patterns. We aimed to investigate the odds of developing severe COVID-19 in health and shift workers.

**Methods:**

We included UK Biobank participants in employment or self-employed at baseline (2006–2010) and with linked COVID-19 data to 31st August 2020. Participants were grouped as neither a health worker nor shift worker (reference category) at baseline, health worker only, shift worker only, or both, and associations with severe COVID-19 investigated in logistic regressions.

**Results:**

Of 235,685 participants (81·5% neither health nor shift worker, 1·4% health worker only, 16·9% shift worker only, and 0·3% both), there were 580 (0·25%) cases of severe COVID-19. The odds of severe COVID-19 was higher in health workers (adjusted odds ratio: 2·32 [95% CI: 1·33, 4·05]; shift workers (2·06 [1·72, 2·47]); and in health workers who worked shifts (7·56 [3·86, 14·79]). Being both a health worker and a shift worker had a possible greater impact on the odds of severe COVID-19 in South Asian and Black and African Caribbean ethnicities compared to White individuals.

**Conclusions:**

Both health and shift work (measured at baseline, 2006–2010) were independently associated with over twice the odds of severe COVID-19 in 2020; the odds were over seven times higher in health workers who work shifts. Vaccinations, therapeutic and preventative options should take into consideration not only health and key worker status but also shift worker status.

**Supplementary Information:**

The online version contains supplementary material available at 10.1186/s12889-021-10839-0.

## Background

The severe acute respiratory syndrome coronavirus 2 (SARS-CoV-2), which causes coronavirus disease-2019 (COVID-19), is a global health threat [1]. It has led to an unprecedented co-ordinated global research effort to develop and evaluate a range of vaccines. To date, preliminary results are in for candidate vaccines and vaccination of priority groups has commenced. The provisional priority list in the UK focuses on care home residents and their carers’, front-line health and social care workers, and older adults [2]. The high priority for health workers and care workers is due to the established elevated odds of infection, development of severe infection, and spreading infection in these groups [3–8]. This is further increased for health workers from ethnic minorities [6]. There has been less attention on whether the odds are exacerbated in those with irregular work patterns, i.e. shift work, which is common in health and care.

Working shifts is associated with an increased risk for cardiovascular disease [9–12] which appears to persist following retirement, although attenuated [12]. Research suggests risk factors for cardiometabolic diseases [12] are also risk factors for COVID-19 [13–15]. Further, shift work is associated with alterations in the immune system and an increased risk for viral infections [16]. In this view, it is not surprising that recent evidence suggests that shift work is associated with elevated odds of severe COVID-19 [5, 17], and that health care workers on night shifts have higher odds of in-hospital SARS-CoV-2 infection than those on day shifts [18]. However, it is not known whether working shifts interacts with health worker status or ethnicity, both of which are independently associated with elevated odds of COVID-19 [6, 19, 20].

Shift workers are more likely to have disturbed sleep and variable sleep patterns [21] leading to disruption of the circadian rhythm. This has been hypothesised to increase the odds of COVID-19 in night shift workers [22], but is evident even if the shift pattern does not include night work, likely due to sleep disruption in relation to circadian rhythms [21], which may persist in the years following cessation of shift work [23]. Recent data have suggested that sleep disruption and high variability in sleep timing are associated with the odds of testing positive for COVID-19 and development of severe infection [15]. Exacerbating this, shift work is common in health workers where exposure to infection with SARS-CoV-2 and odds of developing severe COVID-19 are already relatively high [6, 7]. Therefore, we hypothesise both health workers and shift workers will independently be at increased odds of developing severe COVID-19, but the odds will be further increased in health workers who are also shift workers. Further, we hypothesise that increased odds of developing severe COVID-19 will be evident across ethnic groups and for males and females.

## Methods

This study is reported as per the Strengthening the Reporting of Observational Studies in Epidemiology (STROBE) guidelines (Supplementary material: Checklist S1) and following a pre-specified protocol (Application Number 36371) [24].

### Study population

For this analysis, we used data from UK Biobank (application 36,371), a prospective cohort of > 500,000 adults aged 40–69 years [25]. UK Biobank has full ethical approval from the NHS National Research Ethics Service (16/NW/0274). All methods were carried out in accordance with relevant guidelines and regulations and all participants gave written informed consent prior data collection. All baseline assessments were conducted between March 2006 and July 2010. UK Biobank data are linked to national SARS-CoV-2 laboratory test data through Public Health England’s Second Generation Surveillance System [26]. This secure, pseudonymized, individual-level rapid dynamic linkage is described in detail elsewhere [26]. Data provided by the system is incorporated into the UK Biobank (UKB) database and released through the usual governance processes (http://www.ukbiobank.ac.uk/register-apply). The data were available from 16th March 2020 to 31st August 2020 and included outcome of the test (positive/negative) and specimen origin (hospital inpatient vs other). Analyses were restricted to those who were alive on 16th March 2020 (the first COVID-19 testing sample date) and to English centres as testing data were initially only for those based in England.

#### Exposure

Participants’ occupation was categorised according to whether or not they were health workers and/or shift workers based on the occupation information reported at baseline (2006–2010). Health care workers were classified based on UK Biobank occupational codes 2,211,001–2,216,012. Participants who reported that their work involved shift work “sometimes”, “usually” or “always” were classified as shift workers, while participants who reported that their work “never/rarely” involved shift work were classified as non-shift workers. Shift or health worker status was defined as four mutually exclusive categories: neither (reference category), health worker only, shift worker only, or both health and shift worker. Only participants who reported being in paid employment or self-employed at baseline were asked about shift work. Those without data for shift work and health work status were excluded. A flow chart detailing all participant exclusions is provided in Figure S1 in Supplementary material.

#### Outcome

Severe COVID-19 was defined as a composite of a positive test result for SARS-CoV-2 from a hospital setting in line with guidance for this dataset [26], or death related to the disease (i.e. any death with an ICD-10 code of U07.1 or U07.2 as the primary cause of death on the death certificate). Positive tests in an outpatient setting were removed from this analysis as we were unable to determine whether these ultimately resulted in hospitalisation. Results can thus be interpreted as the overall population level risk of being admitted to hospital with or dying from COVID-19 during the linkage period within UK Biobank. This population level method of assessing risk has been commonly reported within COVID-19 risk factor research, enabling comparison to the literature in terms of how the risk factors assessed compare to other commonly reported risk factors [13–15, 27].

#### Co-variates/confounders

Participant characteristics, including body mass index (BMI), sex, ethnicity (White, South Asian, Black and African Caribbean), deprivation (Townsend score, a composite measure of deprivation based on unemployment, non-car ownership, non-home ownership, and household overcrowding; negative values represent less deprivation), cancer (self-reported), co-morbidities (yes/no; one or more medical condition(s): i.e. cardiovascular, respiratory, renal, neurology, musculoskeletal, haematology, gynaecology, immunology, infections), and smoking status (never, previous current) were collected at the baseline assessment. Age on 16th March 2020 was calculated. Confounders were selected based on current clinical knowledge showing the risk of COVID-19 is elevated in men, ethnic minorities [6], people who are older [2], obese [14], deprived [2], have co-morbidities [2], or who smoke [28].

### Statistical analysis

Logistic regression was used to identify the odds associated with developing severe COVID-19 in participants who were shift workers only, health workers only, or were both health and shift workers. The reference category for comparison was workers who did not work shifts, or work in healthcare. These four categories are mutually exclusive to facilitate interpretation of the independent effects of shift and health worker status, and whether their combination provides an additive or multiplicative association. Analyses were carried out for the whole cohort, and also stratified by ethnicity, and sex. All analyses were adjusted for the aforementioned potential confounders.

Two sensitivity analyses were carried out: 1) with self-reported sleep duration at baseline included as a further co-variate; 2) stratified by retirement age (currently 66 years of age in the UK). People below retirement age at the beginning of the pandemic were assumed most likely to still be working and thus at higher exposure to the virus. People above retirement age were assumed to be less likely to be working and thus at lower exposure to the virus. Individuals with an age at time of COVID-19 test equal to or below 65 years were classed as below retirement age, those with an age at time of COVID-19 above 65 years as above retirement age. Age was measured as an integer in years.

All analyses were carried out in Stata version 16.0 (StataCorp LLC, TX, USA). The code used to run the analysis is available on github (https://github.com/clg13/Employment-analysis-code/blob/main/employment%20analysis%20code%20for%20github.do). Statistical significance was set at the alpha level of .05.

## Results

There were 235,685 participants eligible for inclusion in this analysis (i.e. with information on outcome of severe COVID-19, shift or health worker status, and full co-variate profile), of which 580 (0·25%) had severe COVID-19. Mean participant age was 63·8 years (SD 7·1), BMI 27·2 kg·m^− 2^ (SD 4·7), 52·2% were female, and 96·1% were White (Table 1); 81·5% (*n* = 193,135) were neither a shift nor health worker, 16·9% (*n* = 38,738) were a shift worker only, 1·4% (*n* = 3193) a health worker only, and 0·3% (*n* = 620) both.

**Table 1:** Participant characteristics

Characteristic	All (***n*** = 235,685)	Severe covid-19 (hospitalisation or death)	
		Yes (*n* = 580)	No (n = 235,105)
**Age at test (years)**	63·8 (7·11)	63·6 (7·70)	63·8 (7·10)
**Body mass index (kg/m**^**2**^**)**	27·2 (4·68)	28·7 (5·34)	27·2 (4·68)
**Townsend score**	-1·39 (2·96)	-0·47 (3·39)	-1·39 (2·96)
**Sex**			
Female	123,127 (52·2)	275 (47·4)	122,852 (52·3)
**Ethnicity**			
White	226,436 (96·1)	518 (89·3)	225,918 (96·1)
South Asian	4345 (1·8)	30 (5·2)	4315 (1·8)
Black	4904 (2·1)	32 (5·5)	4872 (2·1)
**Smoking status**			
Never	135,710 (57·6)	300 (51·7)	135,410 (57·6)
Previous	75,767 (32·2)	221 (38·1)	75,546 (32·1)
Current	24,208 (10·3)	59 (10·1)	24,149 (10·3)
**Past or current cancer**			
Yes	13,957 (5·9)	39 (6·7)	13,918 (5·9)
**Co-morbidities**			
Yes	162,290 (68·9)	438 (75·5)	161,852 (68·9)
**Shift or health worker**			
Neither	193,134 (81·5)	375 (64·7)	192,759 (82.0)
Shift worker	38,738 (16·9)	183 (31.6)	38,555 (16·4)
Health worker	3193 (1·4)	13 (2·2)	3180 (1·4)
Both	620 (0·3)	9 (1·6)	611 (0·3)

Values reported are mean (SD) or N (%)

Co-morbidities: yes/no; one or more medical condition(s): i.e. cardiovascular, respiratory, renal, neurology, musculoskeletal, haematology, gynaecology, immunology, infection

Townsend score: a composite measure of deprivation based on unemployment, non-car ownership, non-home ownership, and household overcrowding; negative values represent less deprivation

**Fig. 1:**
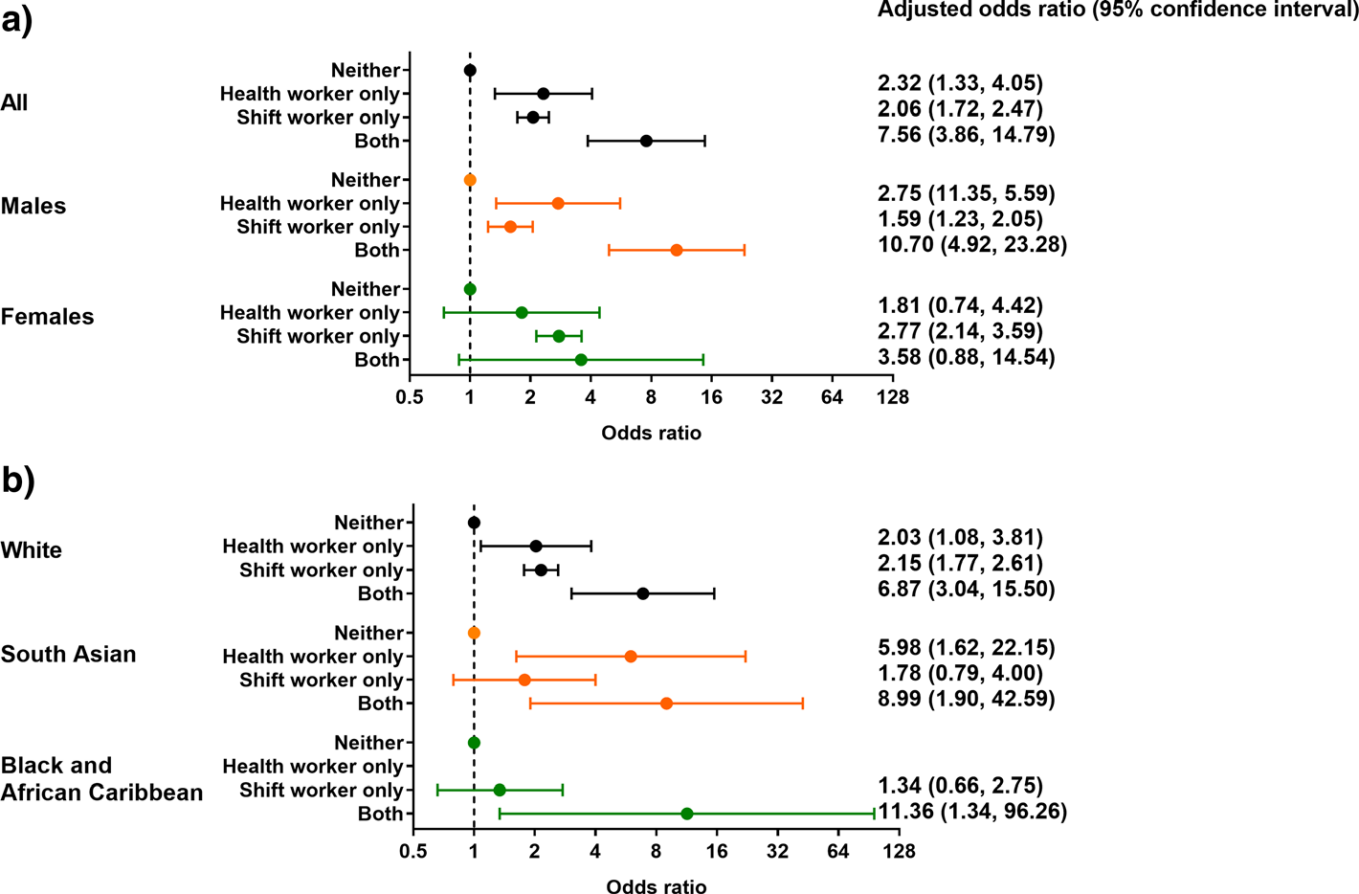
Employment status and odds of severe COVID-19 stratified by **a**) sex, **b**) ethnicity. Model (**a**) adjusted for: age, sex (for all participants), Townsend score, BMI, cancer (self-reported, past or current at time of data collection), co-morbidities (yes/no), smoking status (never, previous current) and ethnicity. Model (**b**) same as model (**a**) except without ethnicity

After adjustment for potential confounders, a significant association was found between shift worker only (adjusted odds ratios (aOR): 2·06 [95% CI: 1·72, 2·47]) or health worker only (2·32 [1·33, 4·05]) status and odds of severe COVID-19 (Fig. 1a). The estimated odds were greatest for individuals who were both a shift and health worker (aOR: 7·56 [3·86, 14·79]). A similar pattern was found when the analysis was stratified by sex (Fig. 1a), with a higher estimated association for both shift and health worker status in men (aOR: 10·70 [4·92, 23·28]) than women (aOR: 3·58 [0·88, 14·54]). When the analysis was stratified by ethnicity (Fig. 1b), there was a tendency for a greater impact of being both a health worker and a shift worker in South Asian and Black and African Caribbean ethnicities when compared to White, but confidence intervals were large.

Results of unadjusted models were consistent with the adjusted models and are given in Supplementary Table S1.

### Sensitivity analyses

In the first sensitivity analysis, controlling for sleep duration did not change the results (Supplementary Figure S2).

**Fig. 2:**
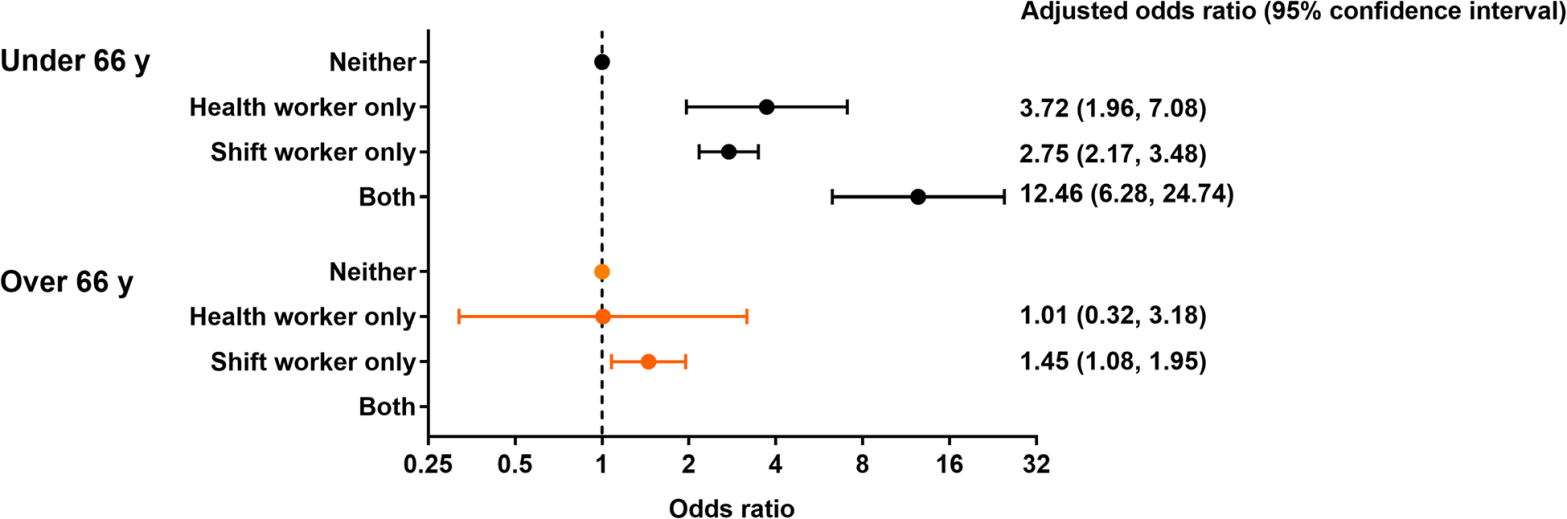
Employment status and odds of severe COVID-19 stratified by UK retirement age (66y). Adjusted for age, sex (for all participants), Townsend score, BMI, cancer (self-reported, past or current at time of data collection), co-morbidities (yes/no), smoking status and ethnicity

The second sensitivity analysis, stratified by retirement age, was conducted for the whole sample only, due to small numbers in the sex and ethnicity sub-groups. There were 125,118 eligible individuals below retirement age (54·2% female, 94·8% White) and assumed to be working, of which 312 (0·25%) had severe COVID-19. Of these 80·1% (*n* = 100,170) were neither a shift nor health worker, 18·2% (*n* = 22,819) were a shift worker only, 1·4% (*n* = 1708) a health worker only, and 0·3% (*n* = 421) both. There was a similar pattern of results, with estimated odds ratios generally larger than when the whole cohort was considered (Fig. 2).

Eligible people above retirement age and assumed to be retired were 110,567 (50·0% female, 97·6% White), of which 268 (0·24%) had severe COVID-19. Of these, 84·1% (*n* = 92,964) had been neither a shift nor health worker, 14·4% (*n* = 15,919) a shift worker only, 1·3% (*n* = 1485) a health worker only, and none who had been both. Elevated odds associated with prior shift worker status persisted, albeit lower (aOR: 1·45 [1·08, 1·95]); conversely, no association with prior health worker status was evident (Fig. 2).

## Discussion

Both being a health worker, or working shifts, were independently associated with over twice the population level odds of severe COVID-19; notably, the odds were more than seven times higher in health workers who work shifts. The impact of health and shift work tends to be higher in males and in minority ethnic groups, who are already at increased risk of severe COVID-19 [19, 20].

The substantially higher odds of severe COVID-19 associated with health workers who work shifts may reflect a greater patient-facing role. This would lead to more viral exposure than non-shift health workers who may be more likely to be in managerial, supervisory or technician roles. The odds of severe COVID-19 were also stronger when considering only people below retirement age at the beginning of the pandemic, thus more likely to still be working and at increased viral exposure. When considering only people above retirement age at the beginning of the pandemic, the association with health worker status appears to dissipate. This potentially suggests that the elevated odds of severe COVID-19 in the whole population or those under retirement age is indeed explained by increased exposure to the virus. In contrast, an association of severe COVID-19 with prior shift-work status persists, although attenuated. Alongside the higher odds for health workers who work shifts, this suggests that the association between severe COVID-19 and shift work may not be fully explained by an increased viral exposure.

The persistence of elevated odds associated with shift work following retirement has previously been identified for cardiovascular disease [12]. Purported mechanisms include disruptions to the behavioural and circadian rhythm [9], which can lead to chronic inflammation [29], potentially contributing to the increased risk of cardiovascular disease observed in previous shift workers. As COVID-19 is an acute inflammatory disease [29], it may exacerbate any existing chronic inflammation. Alongside other risk factors (e.g. health-related behaviours [15], psychological stress and genetic predisposition), this may be associated with a ‘cytokine storm’ [22, 29, 30] contributing to the increased odds of severe COVID-19 we observed in shift-workers.

The demand for 24-h services has extended shift work beyond factories to more traditionally “white collar” occupations, e.g. retail and service [31], with approximately 15–25% of workers in Europe employed on shift schedules [11, 16]. Irrespective, shift workers still tend to be more deprived and subject to psychosocial stresses [10], which may contribute to increased risk for cardiovascular disease and COVID-19. While we controlled for a range of available co-variates, including age, sex, ethnicity, deprivation, co-morbidities and self-reported sleep (sensitivity analysis), other residual confounders, e.g. health-related behaviours, may be present that predispose the shift workers to greater odds of severe COVID-19. However, Maidstone et al. [17] recently showed that the incidence of COVID-19 in shift workers was still greater when compared to non-shift workers in the same job. Further, in a previous UK Biobank study, we showed that objectively measured sleep disruption and variability in sleep timing was associated with increased odds of severe COVID-19 [15]. While disturbed sleep is prevalent in shift workers,^21^ the odds were similar when excluding shift workers from the cohort [15]. This observation would suggest that sleep disturbance and variability in sleep timing, even in the absence of shift work status, is associated with increased odds of severe COVID-19 [32]. Likewise, irregular sleep timing was associated with metabolic abnormalities in a prospective study on cardiovascular events in ~ 2000 participants [33], with similar results when shift workers were excluded.

Strengths of this study include the large population with linked COVID-19 data. In addition, the UK Biobank differs from many other datasets currently being analysed to better understand COVID-19, in that it is an extensively phenotyped population, allowing the impact of issues such as shift worker status to be assessed. However, the study also has several important limitations. Characteristics of participants, including health worker and shift work status, were measured between 2006 and 2010 and may have changed prior to the pandemic. Mutambudzi et al. [5] and Maidstone et al. [17] similarly used occupation at UK Biobank baseline to ascertain odds of severe COVID-19. In support of this assumption, Matambudzi et al. [5] determined a high correlation (r = 0.71, *p* < 0.001) between occupation at baseline and occupation between 2014 and 2019 in a sub-sample of > 12,000, participants indicating a high likelihood that participants had continued working in the same profession. Further, in our analyses stratified by retirement age, we assumed that those below retirement age at the date of their COVID-19 test were still working and at relatively high exposure to COVID-19, while those above retirement age were not working and were at lower exposure. It is not possible to confirm this assumption with the available data. Additionally, the definition of severe COVID-19 was a positive test from a hospital inpatient; while this is consistent with the definition proposed by the researchers that developed the linkage method [25], actual disease severity cannot be confirmed from the linkage data available. Finally, participants in UK Biobank may not be representative of the wider population and testing in the UK has not been universal, making analyses vulnerable to bias.

## Conclusions

In conclusion, both shift and health work status (measured in 2006–2010) were associated with increased odds of developing severe COVID-19 independent of age, sex, ethnicity, deprivation and co-morbidities. The odds were compounded more than three-fold further in health workers who work shifts, irrespective of sex or ethnicity, compared to neither health nor shift worker. The impact of health and shift work tended to be higher in minority ethnic groups, who are already at increased risk of severe COVID-19. The UK Reach study (https://uk-reach.org/main/) will investigate how, and why, ethnicity affects COVID-19 outcomes in healthcare workers. Notably, the elevated odds associated with health workers was no longer apparent in people over retirement age, suggesting that the increased odds are likely explained by the exposure to the virus inherent to the occupation. However, in shift workers, elevated albeit attenuated odds were still evident in people over retirement age, suggesting that the elevated odds associated with shift work may not be fully explained by increased exposure to the virus. This is consistent with previous reports of elevated risk of cardiovascular disease in former shift workers [12] and further supports that risk factors for cardiovascular and cardiometabolic disease are also risk factors for COVID-19 [13–15]. Vaccination, therapeutic and prevention programmes are being prioritised for health care workers. Our data suggest that shift workers should also be prioritised for these preventive measures.

## Supplementary Information

**Additional file 1: Checklist S1.** Strengthening the Reporting of Observational Studies in Epidemiology (STROBE). **Figure S1**. Flow chart of participants included in main analysis. **Figure S2**. Association between employment status and odds of severe COVID-19, stratified by a) sex and b) ethnicity, additionally controlled for self-reported sleep duration. Model (a) adjusted for: age, sex (for all participants), Townsend score, BMI, cancer (self-reported, past or current at time of data collection), co-morbidities (yes/no), smoking status (never, previous current), ethnicity, and self-reported sleep duration. Model (b) same as model (a) except without ethnicity. **Table S1**. Unadjusted Associations between employment status and odds of severe COVID-19.

## Data Availability

This research has been conducted using the UK Biobank Resource under Application 36371. The database supporting the conclusions of this article is available from UK Biobank project site, subject to registration and application process. Further details can be found at https://www.ukbiobank.ac.uk.
