# Supplementary material for: Association of working shifts, inside and outside of healthcare, with severe COVID−19: an observational study
Source: BMC Public Health. 2021 Apr 22;21:773. doi: 10.1186/s12889-021-10839-0 (PMC8061465; doi:10.1186/s12889-021-10839-0)
Supplement: Supplementary file 1 — Additional file 1: Checklist S1. Strengthening the Reporting of Observational Studies in Epidemiology (STROBE). Figure S1. Flow chart of participants included in main analysis. Figure S2. Association between employment status and odds of severe COVID-19, stratified by a) sex and b) ethnicity, additionally controlled for self-reported sleep duration. Model (a) adjusted for: age, sex (for all participants), Townsend score, BMI, cancer (self-reported, past or current at time of data collection), co-morbidities (yes/no), smoking status (never, previous current), ethnicity, and self-reported sleep duration. Model (b) same as model (a) except without ethnicity. Table S1. Unadjusted Associations between employment status and odds of severe COVID-19. [file 12889_2021_10839_MOESM1_ESM.pdf]

Association of working shifts, inside and outside of healthcare, with risk of severe COVID-19: An observational study

A.V. Rowlands, PhD[1,2], C. Gillies PhD[3], Y. Chudasama PhD[3], M.J. Davies MD[1,2], N. Islam PhD[5], D.E. Kloecker MPhil[1,3,6], C. Lawson PhD[3], M. Pareek PhD[7], C. Razieh MSc[1,2], F. Zaccardi PhD[1,3], *T. Yates PhD[1,2], *K. Khunti PhD[1,3,4]

*Joint senior authors

[1] Diabetes Research Centre, Leicester Diabetes Centre, Leicester General Hospital Gwendolen Rd, Leicester, LE5 4PW, UK

[2] National Institute for Health Research (NIHR) Leicester Biomedical Research Centre (BRC), Leicester General Hospital, Leicester, LE5 4PW, UK.

[3] Leicester Real World Evidence Unit, Leicester Diabetes Centre, Leicester General Hospital Gwendolen Rd, Leicester, LE5 4PW, UK

[4] NIHR Applied Research Collaboration – East Midlands (ARC-EM), Leicester General Hospital, Leicester, UK.

[5] Nuffield Department of Population Health, University of Oxford, Oxford, UK

[6] St George's University of London, Tooting, London, UK

[7] Department of Respiratory Sciences, University of Leicester, Leicester, UK

Corresponding author: Alex Rowlands; Diabetes Research Centre, Leicester Diabetes Centre, Leicester General Hospital Gwendolen Rd, Leicester, LE5 4PW, UK. Email: alex.rowlands@le.ac.uk.

Tel: 0116 258 8632

Checklist S1. Strengthening the Reporting of Observational Studies in Epidemiology (STROBE)

	Item No.	Recommendation	Page No.
**Title and abstract**	1	(*a*) Indicate the study's design with a commonly used term in the title or the abstract	Title
		(*b*) Provide in the abstract an informative and balanced summary of what was done and what was found	Abstract
**Introduction**			
Background/rationale	2	Introduction	Introduction, paragraphs 1-3
Objectives	3	State specific objectives, including any prespecified hypotheses	Introduction, paragraph 3
**Methods**			
Study design	4	Present key elements of study design early in the paper	Methods, study population
Setting	5	Describe the setting, locations, and relevant dates, including periods of recruitment, exposure, follow-up, and data collection	Methods, study population
Participants	6	(*a*) *Cohort study*—Give the eligibility criteria, and the sources and methods of selection of participants. Describe methods of follow-up	Methods, study population, exposure, outcome
		(*b*) *Cohort study*—For matched studies, give matching criteria and number of exposed and unexposed	N/A
Variables	7	Clearly define all outcomes, exposures, predictors, potential confounders, and effect modifiers. Give diagnostic criteria, if applicable	Methods, exposure, outcome
Data sources/ measurement	8*	For each variable of interest, give sources of data and details of methods of assessment (measurement). Describe comparability of assessment methods if there is more than one group	Methods, exposure, outcome, co-variates/confounders
Bias	9	Describe any efforts to address potential sources of bias	Statistical analysis, sensitivity analyses
Study size	10	Explain how the study size was arrived at	Supplementary information, Figure S1

Continued on next page

Quantitative variables	11	Explain how quantitative variables were handled in the analyses. If applicable, describe which groupings were chosen and why	Methods, statistical analysis
Statistical methods	12	(*a*) Describe all statistical methods, including those used to control for confounding	Methods, statistical analysis
		(*b*) Describe any methods used to examine subgroups and interactions	Methods, statistical analysis, sensitivity analyses
		(*c*) Explain how missing data were addressed	Methods, statistical analysis
		(*d*) *Cohort study*—If applicable, explain how loss to follow-up was addressed	N/A
		(*e*) Describe any sensitivity analyses	Methods, statistical analysis, sensitivity analyses
**Results**			
Participants	13*	(a) Report numbers of individuals at each stage of study—eg numbers potentially eligible, examined for eligibility, confirmed eligible, included in the study, completing follow-up, and analysed	Methods, study population
		(b) Give reasons for non-participation at each stage	Methods, study population
		(c) Consider use of a flow diagram	Supplementary information, Figure S1
Descriptive data	14*	(a) Give characteristics of study participants (eg demographic, clinical, social) and information on exposures and potential confounders	Results paragraph 1, Table 1
		(b) Indicate number of participants with missing data for each variable of interest	Supplementary information, Figure S1
		(c) *Cohort study*—Summarise follow-up time (eg, average and total amount)	N/A
Outcome data	15*	*Cohort study*—Report numbers of outcome events or summary measures over time	Results, paragraphs 2-3, Table 1
Main results	16	(*a*) Give unadjusted estimates and, if applicable, confounder-adjusted estimates and their precision (eg, 95% confidence interval). Make clear which confounders were adjusted for and why they were included	Results paragraphs 3-5, Figures 1, 2, Supplementary information Table S1 and Figure S2,
		(*b*) Report category boundaries when continuous variables were categorized	N/A
		(*c*) If relevant, consider translating estimates of relative risk into absolute risk for a meaningful time period	N/A

Continued on next page

Other analyses	17	Report other analyses done—eg analyses of subgroups and interactions, and sensitivity analyses	Results, sensitivity analyses Figure 2, Supplementary information Figure S2
**Discussion**			
Key results	18	Summarise key results with reference to study objectives	Discussion paragraph 1
Limitations	19	Discuss limitations of the study, taking into account sources of potential bias or imprecision. Discuss both direction and magnitude of any potential bias	Discussion paragraph 5
Interpretation	20	Give a cautious overall interpretation of results considering objectives, limitations, multiplicity of analyses, results from similar studies, and other relevant evidence	Discussion paragraphs 5-6
Generalisability	21	Discuss the generalisability (external validity) of the study results	Discussion, paragraph 5
**Other information**			
Funding	22	Give the source of funding and the role of the funders for the present study and, if applicable, for the original study on which the present article is based	End of manuscript

*Give information separately for cases and controls in case-control studies and, if applicable, for exposed and unexposed groups in cohort and cross-sectional studies.

Note: An Explanation and Elaboration article discusses each checklist item and gives methodological background and published examples of transparent reporting. The STROBE checklist is best used in conjunction with this article (freely available on the Web sites of PLoS Medicine at http://www.plosmedicine.org/, Annals of Internal Medicine at http://www.annals.org/, and Epidemiology at http://www.epidem.com/). Information on the STROBE Initiative is a

Figure S1: Flow chart of participants included in the study

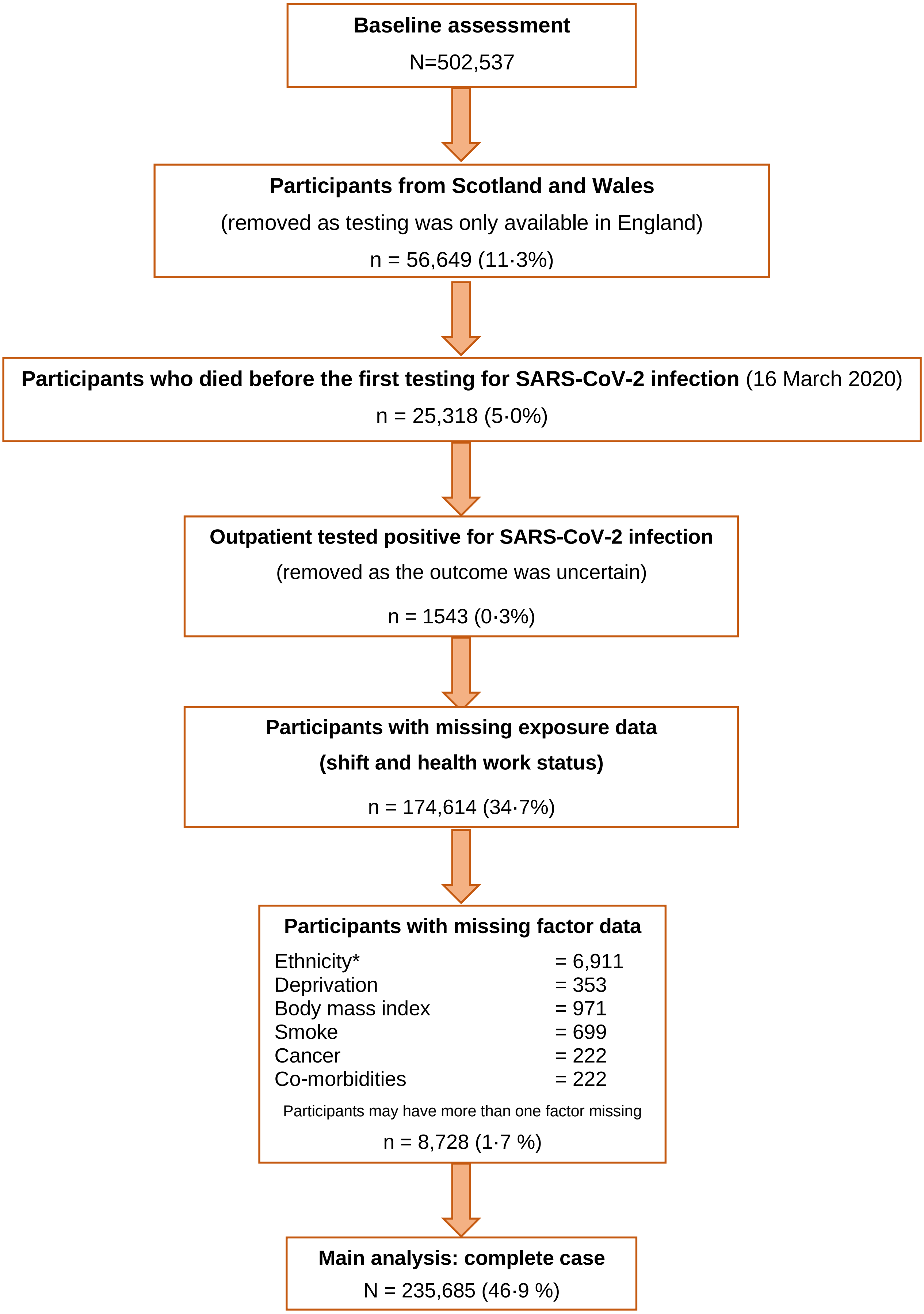

* White Ethnicity, South Asian, Black and African Caribbean

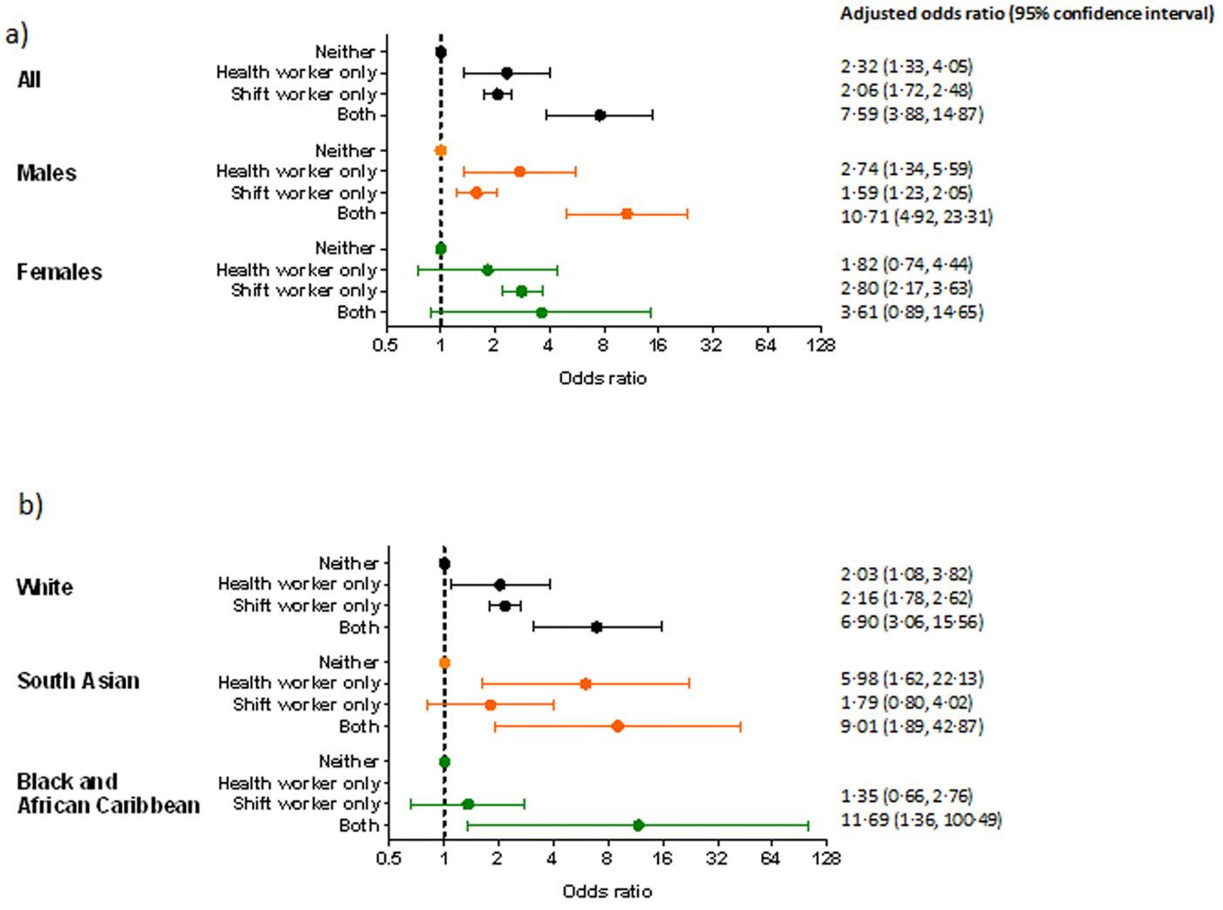

Figure S2. Association between employment status and odds of severe COVID-19, stratified by a) sex and b) ethnicity, additionally controlled for self-reported sleep duration.

Table S1. Unadjusted Associations between employment status and odds of severe COVID-19 (Odds ratio (95% confidence interval))

**Main analysis**	**All**	**Men**	**Women**
Severe covid-19			
Neither	Reference	Reference	Reference
Health worker only	1·99 (1·14, 3·46)	2·43 (1·20, 4·93)	1·56 (0·64, 3·81)
Shift worker only	2·45 (2·06, 2·90)	1·85 (1·45, 2·36)	3·26 (2·56, 4·15)
Both	7·79 (4·14, 14·66)	11·56 (5·70, 23·81)	3·34 (0·83, 13·53)
	**White European**	**South Asian**	**Black and African Caribbean**
Severe covid-19			
Neither	Reference	Reference	Reference
Health worker only	1·80 (0·96, 3·38)	4·60 (1·31, 16·19)	-
Shift worker only	2·41 (1·99, 2·90)	1·96 (0·89, 4·32)	1·60 (0·79, 3·24)
Both	6·13 (2·72, 13·80)	7·14 (1·59, 32·10)	

**Under 66 y**	**All**
Severe covid-19	
Neither	Reference
Health worker only	3·24 (1·71, 6·14)
Shift worker only	3·12 (2·49, 3·91)
Both	13·17 (6·92, 25·06)
**Over 66 y**	**All**
Severe covid-19	
Neither	Reference
Health worker only	0·88 (0·28, 2·74)
Shift worker only	1·78 (1·35, 2·35)
Both	-
